# Experimental Study on Preparation of Inorganic Fibers from Circulating Fluidized Bed Boilers Ash

**DOI:** 10.3390/ma17153800

**Published:** 2024-08-01

**Authors:** Qingjia Wang, Tuo Zhou, Zhiao Li, Yi Ding, Qiang Song, Man Zhang, Nan Hu, Hairui Yang

**Affiliations:** 1 School of Energy and Power Engineering, Changchun Institute of Technology, Changchun 130012, China; wangqingjia@stu.ccit.edu.cn; 2 State Key Laboratory of Power Systems, Department of Energy and Power Engineering, Tsinghua University, Beijing 100084, China; zhoutuo@tsinghua.edu.cn (T.Z.); dingyi@sdic.com.cn (Y.D.); qsong@mail.tsinghua.edu.cn (Q.S.); zhangman@mail.tsinghua.edu.cn (M.Z.); 3 SDIC Power Holdings Co., Ltd., Beijing 100034, China; lizhiao@sdic.com.cn

**Keywords:** circulating fluidized bed ash, inorganic fiber, melting characteristics, viscosity–temperature characteristics, mechanical property

## Abstract

The ash generated by Circulating Fluidized Bed (CFB) boilers is featured by its looseness and porosity, low content of glassy substances, and high contents of calcium (Ca) and sulfur (S), thus resulting in a low comprehensive utilization rate. Currently, the predominant treatment approach for CFB ash and slag is stacking, which may give rise to issues like environmental pollution. In this paper, CFB ash (with a CaO content of 7.64% and an SO_3_ content of 1.77%) was used as the main raw material. The high-temperature melting characteristics, viscosity–temperature characteristics, and initial crystallization temperature of samples with different acidity coefficients were investigated. The final drawing temperature range of the samples was determined, and mechanical property tests were conducted on the prepared inorganic fibers. The results show that the addition of dolomite powder has a significant reducing effect on the complete liquid phase temperature. The final drawing temperatures of the samples with different acidity coefficients range as follows: 1270–1318 °C; 1272–1351 °C; 1250–1372 °C; 1280–1380 °C; 1300–1382 °C; and 1310–1384 °C. The drawing temperature of this system is slightly lower than that of basalt fibers. Based on the test results of the mechanical properties of inorganic fibers, the Young’s modulus of the inorganic fibers prepared through the experiment lies between 55 GPa and 74 GPa, which basically meets the performance requirements of inorganic fibers. Consequently, the method of preparing inorganic fibers by using CFB ash and dolomite powder is entirely feasible.

## 1. Introduction

Circulating fluidized bed (CFB) combustion, a highly efficient combustion technology that can burn inferior fuels such as coal slime, sludge, oil shale, petroleum coke, and coal gangue, has garnered significant attention because of its fuel flexibility, high combustion efficiency, and low pollutant emissions [1,2]. However, CFB boilers mainly burn low-quality coal with high ash content, which will produce a lot of ash during the combustion process [3], with a production of about 300 million tons in China in 2022. Most ash is mainly disposed of by stacking, leading to issues such as occupying farmland [4,5], environmental pollution [6], and ecological damage [7,8]. Therefore, the resource utilization of CFB ash is becoming increasingly urgent. Research on CFB ash utilization started late, but a large number of scholars have conducted related research in recent years. Du et al. [9] optimized the fineness, water requirement ratio, and strength activity index of CFB fly ash by grinding to prepare cement admixture. Cui et al. [10] utilized CFB ash to prepare geopolymers. Lin et al. [11] utilized CFB ash instead of fine bone meal to test the change in mechanical properties. Xia et al. [12] mixed CFB ash and cement to prepare grouting filling materials, and the effects of mixing ratio and water–solid ratio on its fluidity and mechanical properties were studied. Ma and Shoppert et al. [13,14] extracted metal elements from CFB ash for recycling. At present, the utilization of resources is mainly in the field of construction. However, to reduce the emission of NOx, the combustion temperature in the circulating fluidized bed boiler is typically controlled within the range of 850 °C to 900 °C, which is much lower than the combustion temperature of the traditional pulverized coal furnace at 1300–1400 °C. This gives rise to the circulating fluidized bed ash possessing loose and porous traits and a low content of vitreous. Hence, when it is utilized as a building material, a higher water content is demanded. Furthermore, to meet the emission requirements of SO_2_, during the operation of the boiler, desulfurizing agents are injected into the furnace. The typical desulfurizing agent is limestone or dolomite (mainly CaCO_3_). CaCO_3_ decomposes into CaO and CO_2_ at high temperatures. Fresh CaO contacts with SO_2_ to form a sulfur fixation reaction. Since the CaSO_4_ layer formed on the surface prevents the internal CaO from continuing to react, to improve the desulfurization efficiency in the furnace, the calcium–sulfur ratio for desulfurization is generally controlled over 1.2. This part of calcium oxide does not participate in the reaction and becomes free calcium oxide, f-CaO. This leads to a high content of sulfur (S) and free calcium oxide (f-CaO) in the CFB ash [15]. Due to CaO being enveloped by sulfate, it cannot be fully hydrated during material preparation. Subsequent use of the material triggers hydration reactions producing Ca(OH)_2_, and further rehydration reactions with active SiO_2_, and Al_2_O_3_, leading to volume changes which in turn reduces the strength of the construction material. S also generates ettringite during the use of the material leading to changes in volume and affecting the strength of the building material. Therefore, the utilization rate of CFB ash is still very low [16].

Basalt fiber is an inorganic fiber made from natural basalt through high-temperature melting, homogenization, and drawing processes. Due to its excellent tensile strength, high-temperature resistance, corrosion resistance, high Young’s modulus, and other characteristics [17,18,19], basalt fiber has attracted widespread attention and is listed as one of the four key high-performance fibers supported for development in China [20]. However, basalt is widely distributed in nature, and the chemical composition and mineral composition of basalt vary significantly in different regions [21,22,23], leading to the instability of basalt fiber properties. Furthermore, the large-scale mining of basalt has had a serious impact on the ecological environment. The main chemical composition of industrial solid waste like CFB ash is similar to raw materials used in the production of inorganic fibers such as basalt, with SiO_2_, Al_2_O_3_, CaO, and MgO as the primary components. Theoretically, producing inorganic continuous fibers from industrial solid waste is feasible. Some scholars have studied the preparation of inorganic fibers from industrial solid waste. Ma et al. [24] produced inorganic fibers by melting a mixture of 45% pulverized coal furnace ash and waste glass, and the tensile strength of the fibers reached 420 MPa; Kim et al. [25] prepared inorganic fibers using gold tailings, waste limestone, red mud, and nickel–iron ore with Young’s modulus between 60 and 80 GPa, which meets commercial fiber standards. Wen et al. [26] prepared inorganic fibers with high-temperature resistance by mixing pulverized coal furnace ash and magnesium slag, and their tensile strength reached 900 MPa. Through the preparation of inorganic fibers with CFB ash, on the one hand, the resource utilization rate of the CFB ash can be significantly improved, and issues such as ecological damage resulting from stacking and treatment can be alleviated. On the other hand, the inorganic fibers made from CFB ash can bring extra revenues to power plants.

This paper focuses on problems such as environmental pollution and the low utilization rate of CFB ash at present, and combines the preparation process of inorganic fibers, innovatively presenting the process of preparing inorganic fibers using CFB ash. While improving the resource utilization rate of CFB ash and alleviating the environmental pollution problem caused by stacking and disposal, the prepared inorganic fibers bring additional income to the power plant. The melting mechanism of the samples was analyzed through experiments and simulations. The viscosity and initial crystallization temperature of the samples were analyzed through experiments and simulations to determine the final drawing temperature range of samples with different acidity coefficients. The fiber preparation experiment was conducted on the samples, and their macroscopic mechanical properties were analyzed. The feasibility of the inorganic fibers of CFB ash was verified, providing a new way for the resource utilization of CFB ash.

## 2. Research Methods

This paper involved blending CFB ash with dolomite and analyzing their chemical compositions. The simulation analysis of high-temperature melting characteristics and viscosity–temperature characteristics were analyzed by the FactSage software (6.3) package, and the initial crystallization temperature of the glass products after high-temperature melting and homogenization of samples with different acidity coefficients was analyzed by DSC; on this basis, the final drawing temperature range was determined. In addition, fiber preparation experiments were conducted on the samples, and the mechanical properties of the inorganic fibers prepared were tested to validate their feasibility.

### 2.1. Raw Materials

**Figure 1:**
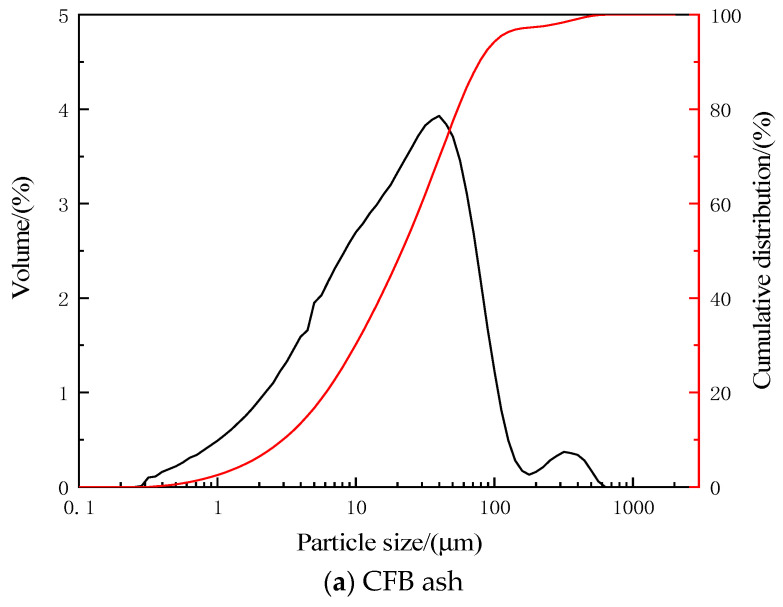

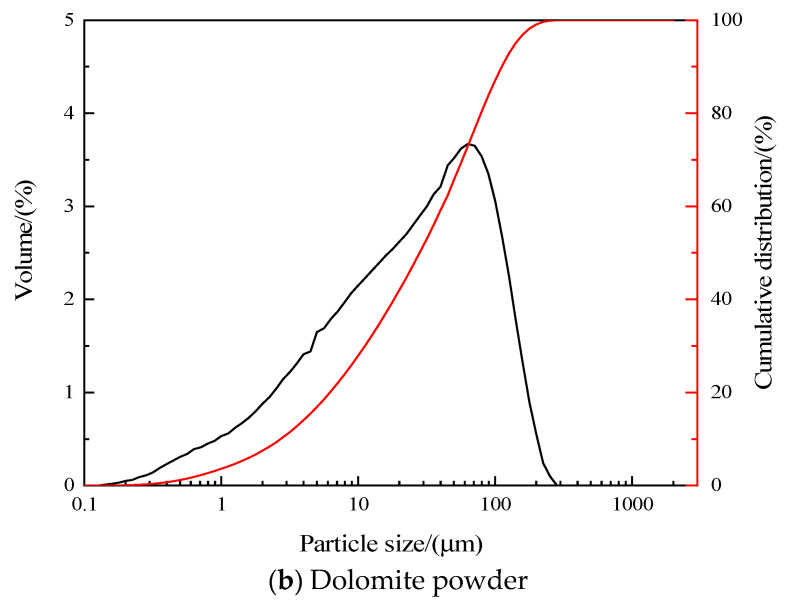
Material particle size distribution.

The CFB ash used in this study was from a 300 MW CFB boiler (The combustion temperature of the boiler was 887 °C. When the CFB ash was collected, the power generation load of the boiler was 217 MW. The fuel used in the boiler was a mixture of coal slime and dry coal). The dolomite powder was purchased from Shanlin Shiyu Mineral Products Co., Ltd. in Guzhang County, China. The particle size distribution of CFB ash and dolomite powder is shown in Figure 1. The acidity coefficient (Mk) is a crucial index for assessing inorganic fibers. In this paper, samples were prepared in accordance with the acidity coefficient (Mk) of inorganic fibers, where Mk = (SiO_2_ + Al_2_O_3_)/(CaO + MgO)(mass ratio), and six samples were mixed as Mk = 2.0, Mk = 2.6, Mk = 3.2, Mk = 3.8, Mk = 4.4, and Mk = 5.0. The chemical compositions of the CFB ash and the six samples are shown in Table 1.

### 2.2. Experimental Process

**Figure 2:**
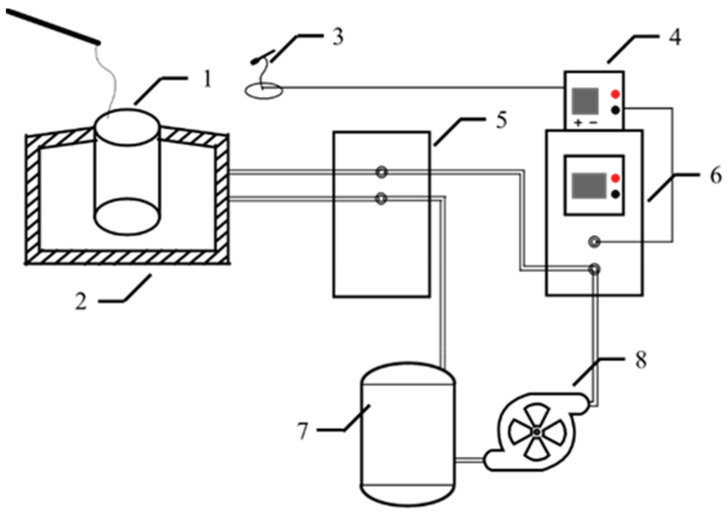
A schematic diagram of the lab bench. 1—graphite crucible; 2—medium frequency induction melting furnace; 3—infrared temperature camera; 4—infrared temperature indicator; 5—water and electricity tank; 6—voltage box; 7—water storage bucket; 8—water pump.

A schematic diagram of the lab bench is shown in Figure 2. The sample mixed with CFB ash and dolomite powder was put into the graphite crucible (radius = 100 mm, height = 200 mm) of the medium frequency induction melting furnace (ZP-25 kW), and it was heated up to the complete liquid phase temperature of the sample to be melted, and then it was kept at this temperature for a minimum of 3 h to make the sample homogenized completely. The temperature of the melts was reduced to the drawing temperature range, and a metal rod was used to perform the drawing operation on the melts in the graphite crucible to thereby obtain inorganic continuous fibers. The remaining melts in the graphite crucible were taken out and cooled to room temperature in the air, and it was milled to be tested for the initial crystallization temperature.

By the mechanical property testing method stipulated in the national standard (GB/T 14337-2022) [27], a fully-automated single fiber universal tester (FAVIMAT) was employed to test the mechanical properties of the prepared inorganic fibers, and the tensile rate was set at 10 mm/min.

### 2.3. Characterization of Microstructure

The chemical composition of CFB ash and six samples with different Mk were tested by X-ray fluorescence spectrometry (XRF-1800, Shimadzu Corporation, Kyoto City, Japan). The particle size analysis of CFB ash and dolomite powder was conducted using a laser particle size analyzer (mastor2000, Malvern Panalytical, Malvern City, UK). The mineral crystal phase of the prepared inorganic fibers was analyzed by X-ray diffractometer (D/max-2550, Rigaku Corporation, Akishima-shi, Japan). The surface morphology of inorganic fibers was analyzed by a high-resolution Zeiss field emission scanning electron microscope (GEMINISEM 500, Carl Zeiss AG, Oberkochen, Germany). The melting and crystallization characteristics of CFB ash and samples with different acidity coefficients were analyzed by using the synchronous thermal analyzer (STA449F3, NETZSCH, Dusseldorf, Germany). The experimental temperature was set at 1600 °C, the heating rate was controlled at 10 °C/min, the atmosphere was air, the gas flow rate was 20 mL/min, the material of the crucible was selected as corundum, and multiple tests were conducted on the samples to ensure the reliability of the experiment.

### 2.4. FactSage Simulation Analysis

In this study, the Equilib module of the FactSage software package was utilized to select FToxid and FactPS databases to calculate the temperature changes of liquid phase content and mineral crystallization in different samples under the temperature range of 800–1600 °C (temperature interval of 50 °C) and air atmosphere. By using the Viscosity module of the FactSage software package and selecting the Melts database, the viscosity of samples with different acidity coefficients (Mk) in the range of 1300–1600 °C (temperature interval of 20 °C) was calculated.

## 3. Results and Discussion

### 3.1. Melting Characteristic Analysis

**Figure 3:**
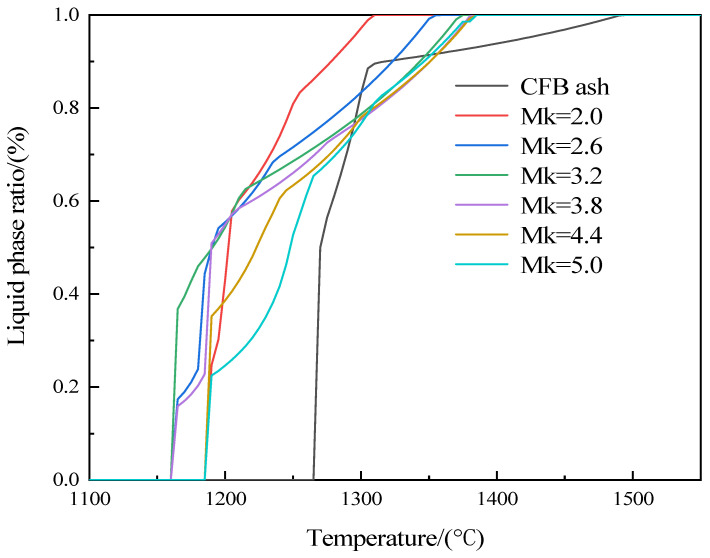
Sample liquid phase ratio and temperature relationship curve.

Material melting homogenization is one of the key processes in the preparation of inorganic fibers. This process ensures that different chemical components in the sample are evenly mixed at high temperatures to form a glassy melt with a uniform microstructure. If the sample is not thoroughly homogenized or not completely melted, the quality of the fibers can be seriously affected. In addition, the melt temperature of the sample directly affects the cost of energy consumption in fiber production. On the premise of guaranteeing melt homogenization, lowering the melt temperature can cut down the production cost, and the addition of dolomite can effectively bring down the melt temperature. Hence, different proportions of dolomite were added to the CFB ash to configure different Mk samples. The variation rule of the liquid phase content of CFB ash and different Mk samples with temperature was calculated by employing the FactSage software package, as shown in Figure 3. The initial liquid phase temperatures and the complete liquid phase temperatures of the CFB ash and different Mk samples are shown in Table 2. Compared to CFB ash, it could be observed that the initial liquid phase formation temperature of CFB ash mixed with dolomite powder samples was significantly lowered. Furthermore, as the Mk decreases, the complete liquid phase temperature continues to decrease. This is the result of decreasing the amount of SiO_2_ and Al_2_O_3_ acidic oxides and an increase in the amount of CaO and MgO alkaline oxides in the sample. In silicate melts, CaO and MgO alkaline oxides act as aiding solvents, and help to reduce the complete liquid phase temperature. On the whole, this system has a lower complete liquid phase temperature compared to natural basalt (1400–1600 °C) [28].

### 3.2. The Mineral Evolution Patterns of Samples with Different Mk with Temperature Changes

**Figure 4:**
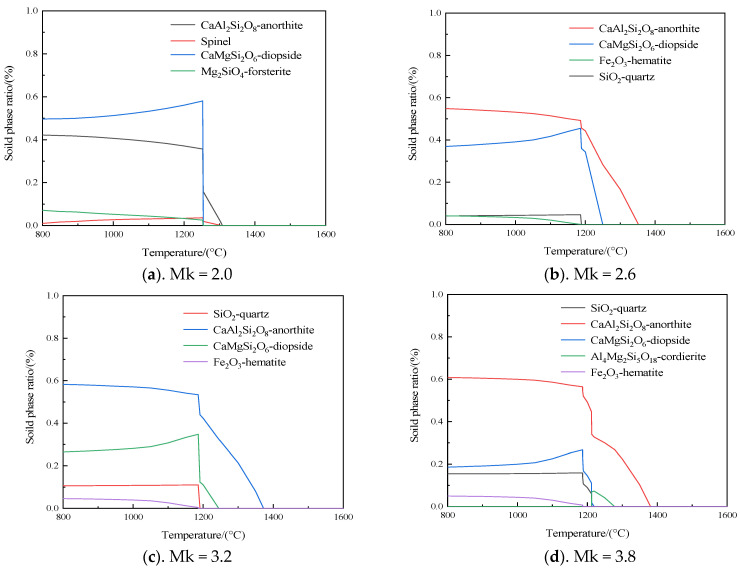

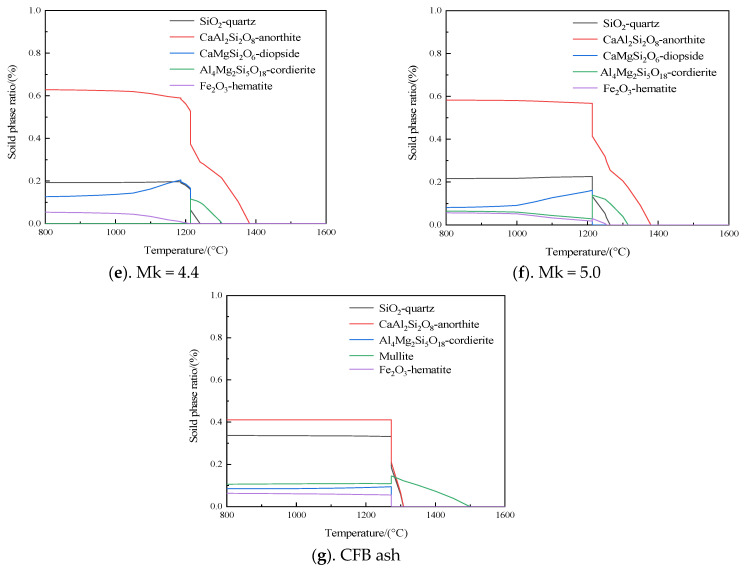
Mineral phase curve of the sample with temperature change.

By using the FactSage software package, the mineral composition after the melting of samples with different mixing proportions was calculated at different temperatures. As shown in Figure 4g, CFB ash at 800 °C mainly contains crystalline phases such as quartz, anorthite, and cordierite. The crystalline phases of various minerals decompose rapidly with the increase in temperature to 1273 °C. The melting point of anorthite is about 1553 °C, and because of its high-temperature instability, it is easy to react with silicate minerals to form low-temperature eutectic substances at high temperatures. Therefore, the content of anorthite gradually decreased with the increase in temperature until it was completely decomposed at 1308 °C. The melting point of quartz is about 1750 °C, but it will react with Al_2_O_3_, SiO_2_, and other low-temperature eutectic substances at 1300 °C, and gradually decompose as the temperature rises. MgO, Al_2_O_3_, and SiO_2_ react to form cordierite with the decomposition of various mineral crystal phases, and its melting point is about 1460 °C. With the gradual increase in temperature, when it reached 1499 °C, accompanied by the decomposition of cordierite, the CFB ash sample was completely liquefied. As shown in Figure 4a–f, the main mineral phases are diopside and anorthite with the Mk decrease, which is related to the gradual decrease in SiO_2_ and Al_2_O_3_ content and the gradual increase in CaO and MgO content. These changes lead to the formation of diopside while reducing the quartz crystal content. The melting point of diopside is about 1390 °C, but it would also participate in the low-temperature eutectic reaction to further reduce the melting point. The above analysis shows that with increasing dolomite powder addition, the SiO_2_ content continuously decreases, while the content of alkaline metal oxides such as CaO and MgO increases. High-temperature mineral phases such as quartz and plagioclase gradually decrease, while the low-temperature eutectic crystal phases such as anorthite and diopside are dominant, which reduces the melting temperature of the whole sample.

### 3.3. High-Temperature Melting Characterization Analysis by TG-DSC

**Figure 5:**
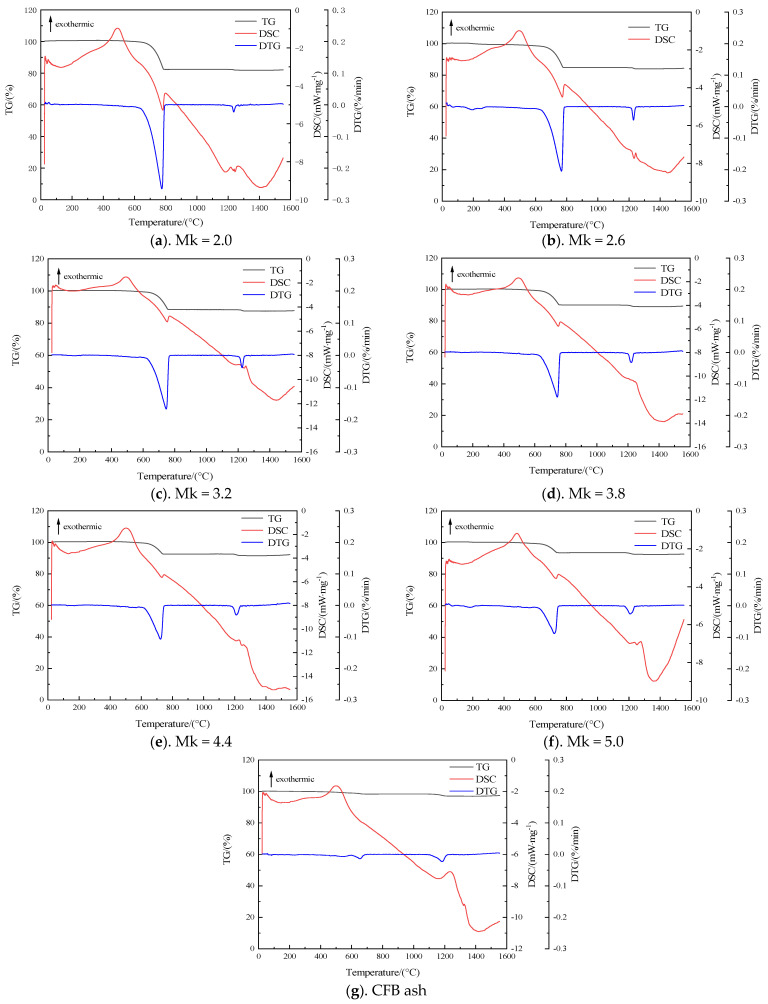
TG-DSC curves of samples with different Mk.

In order to have a clearer understanding of the high-temperature melting process, the samples were analyzed using a simultaneous thermal analyzer (TG-DSC). As shown in Figure 5a–g, with the temperature increasing, the first exothermic peak occurs at 400–600 °C, which is the glass transition temperature, and the atoms in the sample undergo different degrees of rearrangement, resulting in exotherm. The first endothermic peak appears at 700 °C to 750 °C along with a weight loss peak in the TG curve. The mass loss of different samples at this stage is, respectively, 17.62%, 15.20%, 11.6%, 9.88%, 7.35%, 6.43%, and 1.63%. This temperature is the decomposition temperature of carbonate, which generates a large amount of CO_2_ and thus causes the weight loss of the sample. A second endothermic peak is observed at 1150 °C and 1200 °C, with no weight loss on the TG curve, corresponding to the initial liquefaction temperature of the samples analyzed earlier. This temperature represents the temperature at which liquid phase formation occurs. In addition, with decreasing acidity coefficient, the peak gradually becomes steeper, indicating an accelerating liquefaction rate. This corresponds to the results of the analysis of the FactSage software package. A third endothermic peak is observed at 1200 °C to 1253 °C, accompanied by a distinct weight loss peak on the TG curve. The decomposition temperature of CaSO_4_ is about 1200 °C, indicating that this temperature range represents the decomposition temperature of CaSO_4_. The decomposition of CaSO_4_ generates SO_2_ gas, leading to weight loss. The last exothermic peak occurs at 1243–1270 °C, which is the maximum crystallization temperature of the sample. The samples were completely melted at 1380–1440 °C.

### 3.4. Viscosity–Temperature Characterization

**Figure 6:**
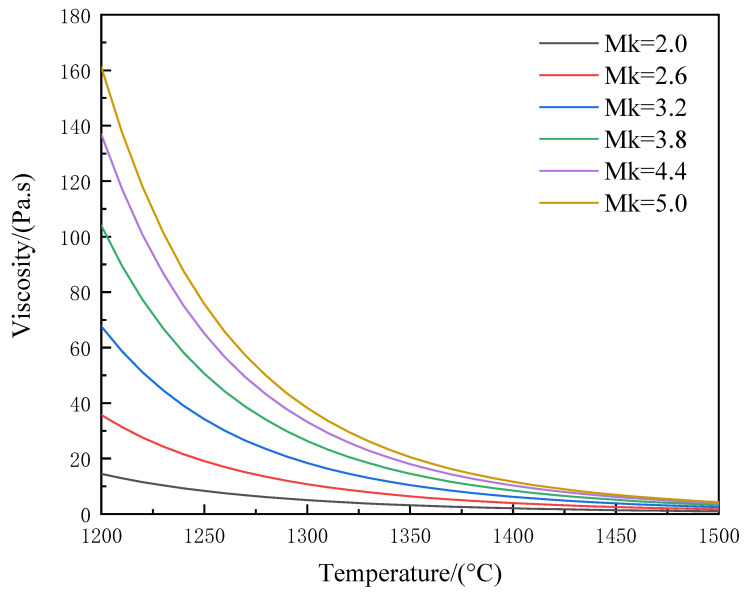
Viscosity of samples at different temperatures.

The viscosity of the melt is critical for the preparation of inorganic fibers. Due to the melt having been fully converted to the liquid phase after melt homogenization, the viscosity of the melt during the cooling process was simulated and analyzed by the FactSage software package. Because the Si/O ratio in the melt is low, as shown in Figure 6, the viscosity of all samples increased with the decrease in temperature, and the temperature range of melt viscosity also decreased with decreasing acidity coefficient, which was related to the addition amount of CaO and MgO. Due to the low Si/O ratio in the melt, the siloxane group in the melt is large and the bond of [SiO]_4_ is strong. The free oxygen generated by CaO and MgO in melting will break the silicon–oxygen network, and alkali metal ions will have a counter-polarization effect on the Si-O bridging oxygen bond, thereby reducing the Si-O bridging oxygen bond strength and reducing the viscosity of the melt.

In the current preparation process of mature inorganic fibers, the range of viscosity is 2.5–30 Pa·s [29]. As shown in Figure 6, in this system, the temperature ranges for different Mk samples that meet the viscosity requirements are 1130–1370 °C, 1210–1460 °C, 1250–1490 °C, 1280–1500 °C, 1300–1500 °C, and 1310–1500 °C, respectively.

### 3.5. Determination of Fiber Drawing Temperature Analysis

**Figure 7:**
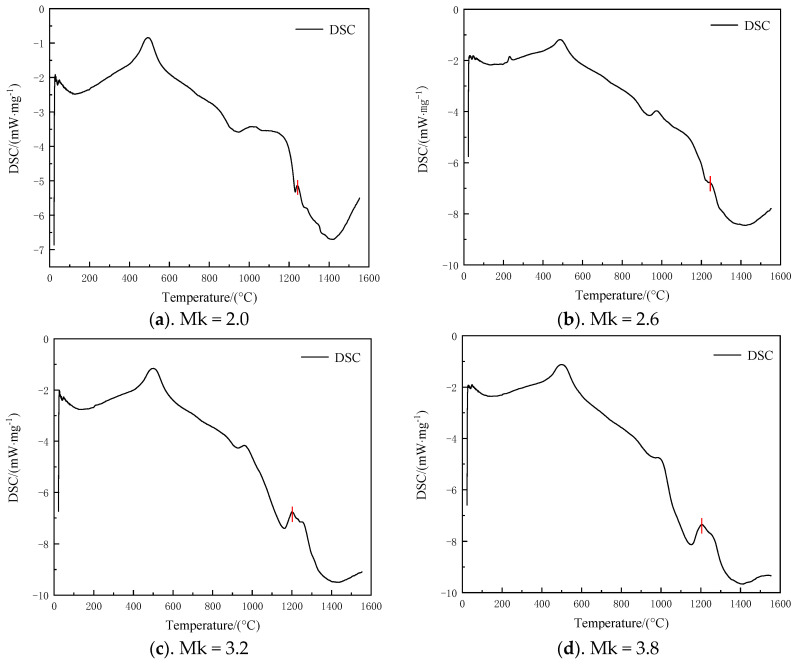

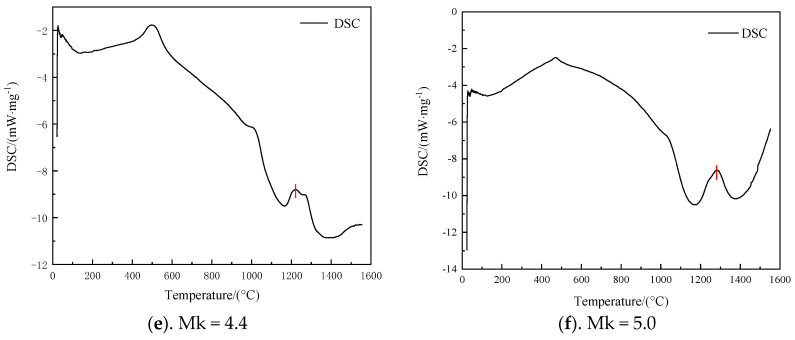
DSC analysis of glassy melt samples with different Mk.

In the drawing process of inorganic fibers preparation, the melt will have different degrees of crystallization tendency, that is, the crystallization characteristics of the melt. Crystallization will reduce the strength of the fiber and even lead to fracture. Therefore, during fiber preparation, the drawing temperature of the fiber must be higher than the initial crystallization temperature of the melt to ensure fiber quality and performance. To determine the initial crystallization temperature more accurately, the sample is transformed into glassy melt following melting and homogenization. Differential scanning calorimetry (DSC) was used in this study to analyze the initial crystallization temperature of the glassy products obtained from high-temperature melting and homogenization of samples with different acidity coefficients. As can be seen from Figure 7, the initial crystallization temperatures of the samples with different acidity coefficients are 1240 °C, 1242 °C, 1201 °C, 1210 °C, 1220 °C, and 1272 °C, respectively. The initial crystallization temperature of basalt fiber is generally 1280 °C [30]. The initial crystallization temperature of this system is slightly lower than that of basalt.

**Figure 8:**
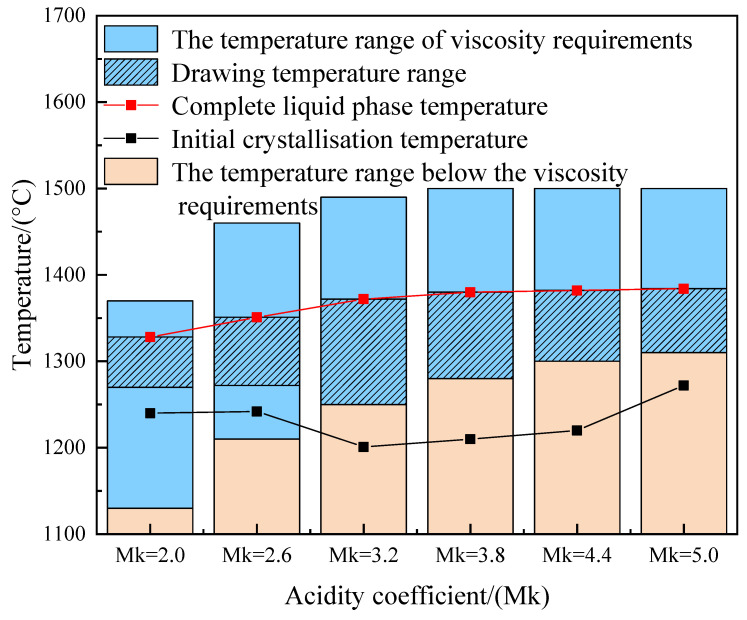
Final fiber drawing temperature range.

Generally, for the quality of inorganic fibers, the drawing temperature of inorganic fibers is higher than the initial crystallization temperature of about 30–40 °C, based on meeting the drawing viscosity requirements. Therefore, the final fiber drawing temperature range of each sample is determined by combining the complete liquid phase temperatures in Figure 3, the temperature ranges of the drawing viscosities in Figure 6, and the initial crystallization temperature in Figure 7. As shown in Figure 8, the final fiber drawing temperature range of different samples can be determined as 1270–1318 °C, 1272–1351 °C, 1250–1372 °C, 1280–1380 °C, 1300–1382 °C, and 1310–1384 °C, respectively. Compared with the current fiber drawing temperature range of basalt fibers (1320–1450 °C), the drawing temperature of this system is slightly lower than that of basalt fiber.

### 3.6. Mechanical Property Analysis

**Figure 9:**
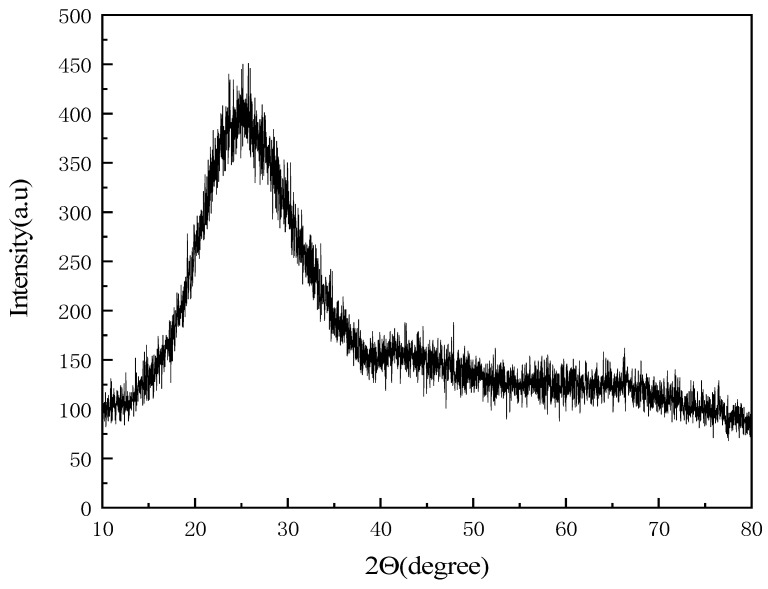
XRD pattern of Mk 2.0 inorganic fiber.

**Figure 10:**
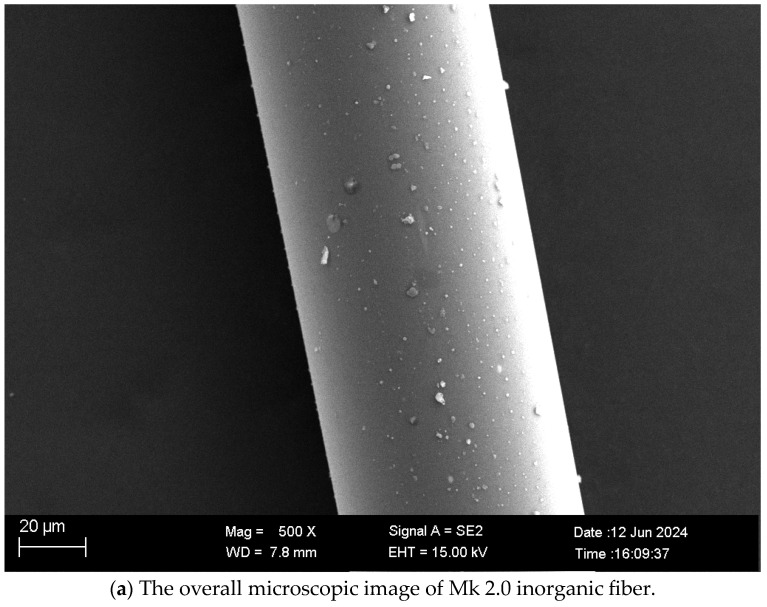

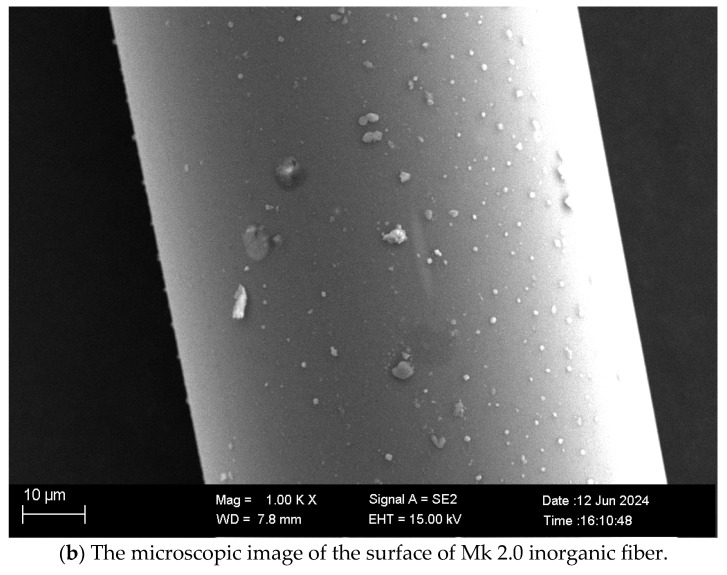
SEM electron microscope image of Mk 2.0 inorganic fiber.

Considering the experimental conditions in the laboratory, when the acidity coefficient was 2.0, the melting temperature and fiber drawing temperature were the lowest; therefore, fiber preparation experiments were carried out with this sample. The X-ray diffraction pattern of the prepared inorganic fiber is shown in Figure 9. There is no diffraction peak of any mineral crystal phase in the prepared inorganic fiber, which proves the accuracy of the fiber drawing temperature range. As shown in Figure 10, the surface characteristics and diameters of inorganic fibers were analyzed by an SEM electron microscope, and the surface of the prepared inorganic fibers is smooth without obvious defects. However, compared to industrially prepared basalt fibers (in the 6 μm to 20 μm diameter range), the currently prepared inorganic fibers are coarser in diameter. The reason is that the fiber drawing rate is low in this experiment. When basalt fibers are prepared for industrial production, the fiber drawing rate is usually controlled between 4 and 12 m/s to obtain a finer fiber diameter. Therefore, adjusting the speed can further optimize the diameter, and the quality of the fiber can be improved by adjusting the fiber drawing rate.

**Figure 11:**
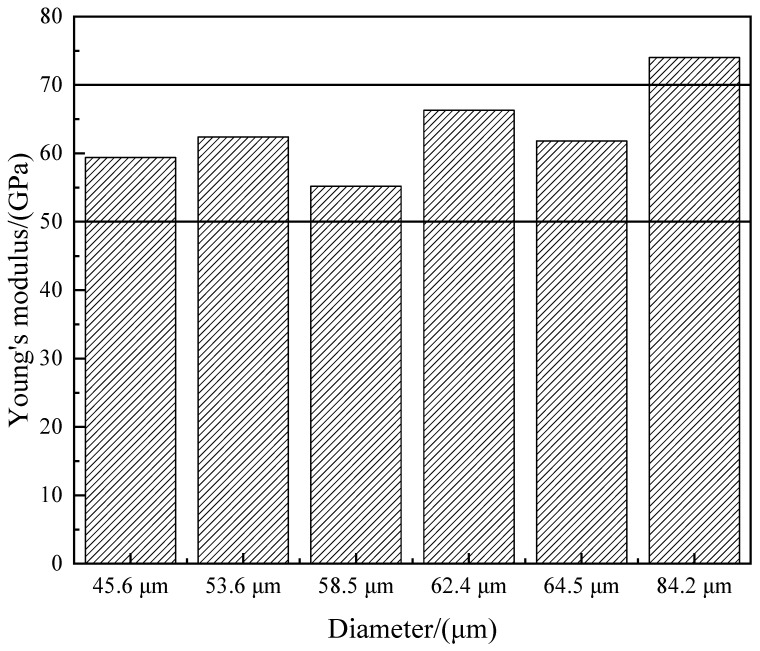
The relationship between Young’s modulus and diameter of inorganic fiber.

The mechanical properties of the fibers were analyzed. As shown in Table 3, the tensile strength of the inorganic fibers prepared in this experiment is in the range of 386 MPa–686 MPa, which is only about one-fourth of the tensile strength of basalt fibers. The increase in fiber diameter leads to a stress concentration effect, internal structural changes, and the appearance of microcracks both inside and outside the fiber. When external forces are applied to the fiber, cracks in the weakest area will quickly propagate, resulting in a brittle fracture of the fiber. Jr Knox et al. [31] also proposed that the tensile strength of the fiber is closely related to the diameter of the fiber. With increasing diameter, the possibility of the combination of larger defects and weaker defects in the fiber is greater; thus, the diameter of the fiber is negatively correlated with the tensile strength. Ren et al. [32] found through the test of different diameters of basalt fibers that the larger the diameter of the fiber, the smaller the stress value, whereas the smaller the diameter of the fiber, the larger the stress value. Therefore, when measuring the diameter of larger inorganic fibers, the strength has a greater degree of variability. Deak et al. [33] studied the mechanical properties of basalt fiber and glass fiber with different diameters. When the diameter of the fiber is less than 9 μm, Young’s modulus tends to become stronger as the diameter decreases, while when the diameter of the fiber is more than 10 μm, Young’s modulus of the fiber is independent of the diameter. Therefore, it is more reasonable to use Young’s modulus to characterize the mechanical properties of fibers. The test results showed that Young’s modulus of the current industrial continuous basalt fiber is between 50 GPa and 70 GPa. As shown in Figure 11, the inorganic fibers prepared by this experiment have Young’s modulus between 55 GPa and 74 GPa and have similar mechanical properties to continuous basalt fiber. At present, the application of basalt fibers mainly focuses on fields such as road construction, filter materials, and high-performance safety protective clothing. The inorganic fibers prepared in this system have similar mechanical properties to basalt fibers. Therefore, it is expected that the inorganic fibers prepared in this system will also have wide applications in fields such as road construction, filter materials, and high-performance safety protective clothing.

## 4. Conclusions

In this paper, inorganic fibers were prepared by compounding dolomite powder with CFB ash, and the complete liquid phase line temperatures, high-temperature melt characteristics, viscosity–temperature characteristics, and initial crystallization temperatures of the samples with different acidity coefficients were analyzed by combining experiments and simulations with FactSage software. The final drawing temperature and suitable boundary conditions were determined. Inorganic fibers were prepared through experiments and their mechanical properties were analyzed. The main conclusions are as follows:

(1) With the continuous decrease in the acidity coefficient, the complete liquid phase temperature of the samples gradually decreases. The high-temperature melting mechanism of the samples was analyzed from the perspective of mineral crystalline phases through TG-DSC. With the gradual decrease in Mk, that is, the content of alkaline metal oxides such as CaO and MgO in the materials gradually increases, high-temperature mineral crystalline phases such as quartz and feldspar gradually decrease, and mineral crystalline phases such as anorthite and diopside that are prone to eutectic reactions gradually dominate, resulting in a decrease in the complete liquid phase temperature of the samples.

(2) The viscosities of samples with different acidity coefficients were analyzed. With the continuous decrease in the acidity coefficient Mk, the temperature range of the melt viscosity is also continuously decreasing. This is related to the added amount of CaO and MgO. Due to the relatively low Si/O ratio in the melt, it causes larger silicon–oxygen anion groups in the melt, and further leads to a high bond strength of [SiO]_4_. The free oxygen generated by the melting of CaO and MgO will break the silicon–oxygen network. The alkaline metal ions Ca^2+^ and Mg^2+^ will have an anti-polarization effect on the Si-O bond in the network, thereby reducing the bond strength of Si-O and reducing the viscosity of the melt.

(3) The final drawing temperature range of samples with different acidity coefficients was determined by the complete liquid phase temperature, the temperature range of melt viscosity, and the initial crystallization temperature. Inorganic fibers were prepared through experiments, and the mechanical properties of the prepared inorganic fibers were measured. The Young’s modulus is in line with the mechanical properties of basalt fibers. Therefore, the inorganic fibers prepared from CFB ash can replace basalt fibers in some fields and have certain economic feasibility. Moreover, the application of basalt fibers mainly focuses on fields such as road construction, filter materials, and high-performance safety protective clothing. Therefore, it is expected that the inorganic fibers prepared in this system will also have wide applications in fields such as road construction, filter materials, and high-performance safety protective clothing.

## Figures and Tables

**Table 1:** Chemical composition of CFB ash and six samples.

(wt%)	SiO_2_	Al_2_O_3_	CaO	Fe_2_O_3_	K_2_O	SO_3_	TiO_2_	MgO	Na_2_O
CFB Ash	54.19	23.64	7.64	5.85	2.50	1.77	1.61	1.08	0.93
Mk = 2.0	41.68	18.07	19.59	4.52	1.91	1.35	1.24	10.29	0.72
Mk = 2.6	44.75	19.44	16.66	4.85	2.05	1.46	1.33	8.03	0.77
Mk = 3.2	46.91	20.40	14.60	5.07	2.15	1.53	1.39	6.44	0.81
Mk = 3.8	48.51	21.11	13.07	5.25	2.23	1.58	1.44	5.26	0.83
Mk = 4.4	49.75	21.66	11.88	5.38	2.29	1.62	1.48	4.35	0.85
Mk = 5.0	50.73	22.10	10.95	5.49	2.33	1.66	1.51	3.62	0.87

**Table 2:** Liquid phase temperature of different samples.

(°C)	Initial Liquid Phase Formation Temperature	Complete Liquid Phase Temperature
CFB Ash	1273.00	1499.00
Mk = 2.0	1253.00	1318.00
Mk = 2.6	1186.00	1351.00
Mk = 3.2	1186.00	1372.00
Mk = 3.8	1186.00	1380.00
Mk = 4.4	1186.00	1382.00
Mk = 5.0	1213.00	1384.00

**Table 3:** Mechanical properties of inorganic fiber.

Diameter/μm	Cross-Sectional Area/m^2^	Tensile Strength/MPa	Young’s Modulus/GPa
45.6	1.63 × 10^−9^	533	59.4
62.4	3.06 × 10^−9^	663	66.3
58.5	2.69 × 10^−9^	386	55.2
53.6	2.26 × 10^−9^	686	62.4
64.5	3.27 × 10^−9^	494	61.8
84.2	5.56 × 10^−9^	469	74.0

## Data Availability

Data are contained within the article.
